# Microbial antibiotics take the lead in the fight against plant pathogens

**DOI:** 10.1111/1751-7915.14185

**Published:** 2022-12-05

**Authors:** Amalia Roca, Miguel A. Matilla

**Affiliations:** ^1^ Department of Microbiology, Facultad de Farmacia Campus Universitario de Cartuja, Universidad de Granada Granada Spain; ^2^ Department of Biotechnology and Environmental Protection, Estación Experimental del Zaidín Consejo Superior de Investigaciones Científicas Granada Spain

## Abstract

The plant microbiome is essential for plant fitness and health. Antibiotics produced by plant‐associated bacteria have been shown to play an important role in protecting plant hosts against phytopathogens. Here, we highlight the strong biotechnological potential of (i) antibiotic producing plant‐associated bacteria as biocontrol agents and (ii) the heterologous expression of antibiotic biosynthetic gene clusters in non‐pathogenic plant‐associated bacteria. We also provide the complete list of the active substances based on bacteria, fungi, and viruses currently approved or pending approval in the European Union, as an indication of the significant emergence and biotechnological applicability of biopesticides. Further progress in this field of research will enable the development of novel biopesticides for the biocontrol of agricultural pests.

## Graphical abstract

The potential of plant microbiome for the development of novel biopesticides for the integrated pest management of agricultural crops.

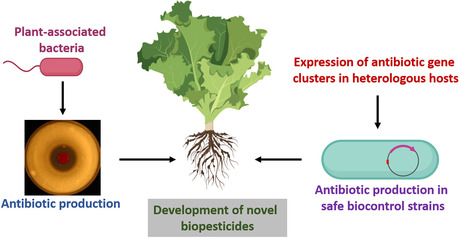

The challenges facing today's society, including climate change, soil and water pollution, and global demographic growth, emphasize the need for urgent changes in our agricultural production system. The plant microbiome, understood as the whole set of microorganisms that establish associations with plants, is key to plant health and productivity and plays a key role in plant survival under biotic and abiotic stresses (Trivedi et al., 2020). Furthermore, it represents the basis of the rational and environmentally sustainable changes that modern agriculture requires. To exploit the potential of plant‐associated microorganisms as biostimulants, biofertilizers, and biopesticides, a deeper knowledge needs to be gained in the assembly of the plant microbial community, plant microbiome responses to biotic and abiotic stresses, as well as the complex interactions between plant‐associated microorganisms and between microbes and their plant hosts (Bazany et al., 2022; Compant et al., 2019; Rico‐Jiménez et al., 2022). Some of these aspects have been addressed in a recent study in *Microbial Biotechnology* where the authors found important differences in the microbial composition and diversity of healthy and diseased plants, providing evidences that specific native microbial members may assist the pythopathogens during the infection process (Xing et al., 2022).

Beneficial plant‐associated bacteria can promote plant growth directly or indirectly by improving nutrient uptake, promoting tolerance to abiotic stresses, synthesizing phytohormones or protecting against plant diseases, among other mechanisms (Compant et al., 2019; Matilla & Krell, 2018; Trivedi et al., 2020). The biocontrol potential of the plant microbiome is the result of a multifactorial process that includes the induction of plant systemic resistance, production of lytic enzymes, competition for nutrients and space, and the biosynthesis of pathogen‐inhibiting volatiles and antibiotics (Carrión et al., 2019; Matilla & Krell, 2018; Trivedi et al., 2020). Given the need to identify new antibiotics with novel mechanisms of action (Krell & Matilla, 2022), it is encouraging that current estimates indicate that only ~3% of the bacterial genetic potential for the synthesis of bioactive compounds has been explored experimentally (Gavriilidou et al., 2022), and plant‐associated biocontrol agents are a promising source for novel antibiotics (Carrión et al., 2019; Gavriilidou et al., 2022).

Where should we look for novel biocontrol agents with new mechanisms of action? Perhaps, a more than realistic response is in suppressive soils (Carrión et al., 2019; Ossowicki et al., 2021) and in ecological niches where the target plant pathogen can be found. Exemplifying this latter case, a study recently published in *Nature Microbiology* analysed the bacterial microbiome of perithecia (sexual fruiting bodies) of *Fusarium graminearum* (Xu et al., 2022) – a fungus ranked in the top 10 of fungal pathogens of relevance in phytopathology (Dean et al., 2012). The authors isolated over 2000 bacterial isolates from more than 60 independent perithecium samples collected from different locations. Among these bacteria, one *Pantoea agglomerans* isolate showed the strongest in vitro inhibition against *F. graminearum* and was found to very efficiently inhibit perithecium formation in several crops of high agronomic interest, including fungal disease progression *in planta* during three consecutive years in field trials conducted on wheat (Xu et al., 2022). Multidisciplinary approaches identified the lipopeptide herbicolin A as the antibiotic responsible for these biopesticidal properties and allowed the identification and characterization of the corresponding antibiotic gene cluster (AGC). Chemical analyses of individual mutants in the genes of the herbicolin A gene cluster enabled the identification of biosynthetic intermediates and propose a model for the synthesis of the herbicolin A (Xu et al., 2022). Through the isolation of fungal‐resistant mutants, the mode of action of herbicolin A was determined. It was shown that ergosterol‐deficient mutants were more resistant to the antifungal antibiotic (Xu et al., 2022). Ergosterol is a key component of fungal cell membranes (Jordá & Puig, 2020), and it was found that herbicolin A specifically interacts with ergosterol as well as with fungal membranes enriched in this sterol lipid. It was demonstrated that herbicolin A disturbs, in vivo and in vitro, the integrity of ergosterol‐containing fungal lipid rafts – microdomains found in cell membranes that are enriched in particular lipids and proteins (Bukrinsky et al., 2020) – to ultimately alter membrane architecture and permeability (Xu et al., 2022). Computational biology approaches showed that herbicolin A was only stably embedded in lipid membranes in the presence of ergosterol, which is, most likely, the reason why the fungi deficient in ergosterol biosynthesis were resistant to herbicolin A. Remarkably, herbicolin A was efficient against fungicide‐resistant *Fusarium graminearum* strains and showed synergistic effects with other fungicides in the treatment of phytopathogens (Xu et al., 2022).

Despite the obvious benefits of microbial‐based biopesticides over chemical pesticides (e.g., reduced release of toxic chemical residues, reduced resistance, low risk of affecting non‐target organisms, and high performance) (Marrone, 2019), the main limitations hindering the definitive take‐off of biopesticides are current legislative restrictions (Dietz‐Pfeilstetter et al., 2021; Marrone, 2019). For example, although the European Union (EU) Green Deal on sustainable control of plant diseases aims to reduce the use and risk of chemical pesticides by 50% in 2030, the complexity of the current EU regulatory framework for the authorization of novel microbial‐based biocontrol agents limits the incorporation of sustainable alternatives to chemical pesticides within the EU (Helepciuc & Todor, 2022). In this regard, among the over 500 active substances currently approved or pending approval in the EU as possible components of pesticide products, around 100 are bacteria, fungi, or viruses that act as insecticides, acaricides, nematicides, fungicides, bactericides, elicitors, or disinfectants. This group of active substances based on microorganisms and viruses includes mainly strains belonging to *Bacillus*, *Pseudomonas*, *Metarhizium*, *Beauveria*, and *Trichoderma* genera (Table 1).

**TABLE 1:** Active substances based on microorganisms (bacteria, fungi, and viruses) approved or pending approval in the European Union as components of pesticide products.

Bacteria/fungi/virus^a^ ^,^ ^b^	Biopesticide type	Status under Reg. (EC) No 1107/2009^c^
*Adoxophyes orana* GV strain BV‐0001	Insecticide	A
*Akanthomyces muscarius* Ve6 (formerly *Lecanicillium muscarium* strain Ve6)	Insecticide	A
*Ampelomyces quisqualis* strain AQ10	Fungicide	A
*Aspergillus flavus* MUCL 54911	Fungicide	P
*Aureobasidium pullulans* (strains DSM 14940 and DSM 14941)	Fungicide, bactericide	A
*Bacillus amyloliquefaciens* (formerly *B. subtilis*) strain QST 713	Fungicide, bactericide	A
*Bacillus amyloliquefaciens* strain AH2	Fungicide	A
*Bacillus amyloliquefaciens* strain AT‐332	Fungicide	P
*Bacillus amyloliquefaciens* strain FZB42	Fungicide	P
*Bacillus amyloliquefaciens* strain IT‐45	Fungicide	A
*Bacillus amyloliquefaciens* MBI 600	Fungicide	A
*Bacillus amyloliquefaciens* strain FZB24	Fungicide	A
*Bacillus amyloliquefaciens* subsp*. plantarum* D747	Fungicide	A
*Bacillus firmus* strain I‐1582	Nematicide	A
*Bacillus licheniformis* strain FMCH001	Fungicide, nematicide	P
*Bacillus nakamurai* strain F727	Fungicide	P
*Bacillus pumilus* strain QST 2808	Fungicide	A
*Bacillus subtilis* strain FMCH002	Fungicide, bactericide	P
*Bacillus subtilis* strain IAB/BS03	Fungicide	A
*Bacillus subtilis* strain RTI477	Fungicide	P
*Bacillus thuringiensis* subsp*. aizawai* strain ABTS‐1857	Insecticide	A
*Bacillus thuringiensis* subsp. *aizawai* strain GC‐91	Insecticide	A
*Bacillus thuringiensis* subsp. *aizawai* strains ABTS‐1857, GC‐91	Insecticide	A
*Bacillus thuringiensis* subsp*. israeliensis* (serotype H‐14) strain AM65‐52	Insecticide	A
*Bacillus thuringiensis* subsp. *kurstaki* strain ABTS 351	Insecticide	A
*Bacillus thuringiensis* subsp*. kurstaki* strain EG 2348	Insecticide	A
*Bacillus thuringiensis* subsp*. kurstaki* strain PB 54	Insecticide	A
*Bacillus thuringiensis* subsp*. kurstaki* strain SA 11	Insecticide	A
*Bacillus thuringiensis* subsp*. kurstaki* strain SA 12	Insecticide	A
*Bacillus thuringiensis* subsp*. Kurstaki* strains ABTS 351, PB 54, SA 11, SA12 and EG 2348	Insecticide	A
*Bacillus velezensis* strain RTI301	Insecticide, bactericide	P
Bacteriophage of Potato Soft Rot *Enterobacteriaceae* (BPSRE)	Bactericide	P
*Beauveria bassiana* strain 203	Insecticide	A
*Beauveria bassiana* strain IMI389521	Insecticide	A
*Beauveria bassiana* strain PPRI 5339	Insecticide	A
*Beauveria bassiana* strain 147	Insecticide	A
*Beauveria bassiana* strain ATCC 74040	Insecticide	A
*Beauveria bassiana* strain GHA	Insecticide	A
*Beauveria bassiana* strain NPP111B005	Insecticide	A
*Beauveria bassiana* strains ATCC 74040 and GHA	Insecticide	A
*Candida oleophila* strain O	Fungicide	A
*Clonostachys rosea* strain J1446 (*Gliocladium catenulatum* strain J1446)	Fungicide	A
*Coniothyrium minitans* strain CON/M/91–08 (DSM 9660)	Fungicide	A
*Cryptophlebia peltastica* nucleopolyhedrovirus strain SouthAfrica	Insecticide	P
*Cydia pomonella* Granulovirus (CpGV)	Insecticide	A
*Fusarium* sp. L13	Fungicide	P
*Helicoverpa armigera* nucleopolyhedrovirus (HearNPV)	Insecticide	A
*Isaria fumosorosea apopka* strain 97 (formerly *Paecilomyces fumosoroseus*)	Insecticide	A
*Metarhizium brunneum* strain BNL102	Insecticide, acaricide	P
*Metarhizium brunneum* strain Cb15‐III	Insecticide	P
*Metarhizium brunneum* strain Ma 43 (formerly *Metarhizium anisopliae* var *anisopliae* strain F52)	Insecticide, acaricide	A
*Metarhizium pingshaense* strain CF62	Insecticide	P
*Metarhizium pingshaense* strain CF69	Insecticide	P
*Metarhizium pingshaense* strain CF78	Acaricide	P
*Metschnikowia fructicola* strain NRRL Y‐27328	Fungicide	A
Mild Pepino Mosaic virus isolate VC 1	Elicitor	A
Mild Pepino Mosaic virus isolate VX 1	Elicitor	A
*Paecilomyces fumosoroseus* strain FE 9901	Insecticide	A
*Pasteuria nishizawae* strain Pn1	Nematicide	A
Pepino mosaic virus (PepMV) Chilean (CH2) strain, mild isolate Abp2 (PEPMVO)	Elicitor	A
Pepino mosaic virus (PepMV) European (EU) strain, mild isolate Abp1 (PEPMVO)	Elicitor	A
Pepino mosaic virus strain CH2 isolate 1906	Elicitor	A
*Phlebiopsis gigantea* strain FOC PG 410.3	Fungicide	A
*Phlebiopsis gigantea* strain VRA 1835	Fungicide	A
*Phlebiopsis gigantea* strain VRA 1984	Fungicide	A
*Phthorimaea operculella* granulovirus (PhopGV)	Insecticide	P
*Pseudomonas chlororaphis* strain MA342	Fungicide	A
*Pseudomonas* sp. strain DSMZ 13134	Fungicide	A
*Purpureocillium lilacinum* strain PL 11	Nematicide	A
*Purpureocillium lilacinum* strain 251 (former *Paecilomyces lilacinus* strain 251)	Nematicide	A
*Pythium oligandrum* strain M1	Fungicide	A
*Pythium oligandrum* strain B301	Fungicide	P
Saccharomyces cerevisiae strain LAS02	Fungicide	A
*Spodoptera exigua* multicapsid nucleopolyhedrovirus (SeMNPV), isolate BV‐0004	Insecticide	A
*Spodoptera littoralis* nucleopolyhedrovirus (SpliNPV)	Insecticide	A
*Streptomyces* K61 (formerly *S. griseoviridis*)	Fungicide	A
*Streptomyces lydicus* WYEC 108	Fungicide	A
*Trichoderma afroharzianum* strain T‐22 (formerly *Trichoderma harzianum* strain T‐22)	Fungicide	A
*Trichoderma afroharzianum* strain Th2RI99	Fungicide	P
*Trichoderma asperellum* (formerly *T. harzianum*) strain ICC012	Fungicide	A
*Trichoderma asperellum* (formerly *T. harzianum*) strain T25	Fungicide	A
*Trichoderma asperellum* (formerly *T. harzianum*) strain TV1	Fungicide	A
*Trichoderma asperellum* (formerly *T. harzianum*) strains ICC012, T25 and TV1	Fungicide	A
*Trichoderma asperellum* strain T34	Fungicide	A
*Trichoderma atrobrunneum* (formerly *T. harzianum*) strain ITEM 908	Fungicide	A
*Trichoderma atroviride* (formerly *T. harzianum*) strain IMI 206040	Fungicide	A
*Trichoderma atroviride* (formerly *T. harzianum*) strain T11	Fungicide	A
*Trichoderma atroviride* (formerly *T. harzianum*) strain T11 and IMI 206040	Fungicide	A
*Trichoderma atroviride* strain 77B	Fungicide, disinfectant	P
*Trichoderma atroviride* strain AGR2	Fungicide, disinfectant	P
*Trichoderma atroviride* strain AT10	Fungicide, disinfectant	P
*Trichoderma atroviride* strain I‐1237	Fungicide	A
*Trichoderma atroviride* strain SC1	Fungicide	A
*Trichoderma gamsii* (formerly *T. viride*) strain ICC080	Fungicide	A
*Trichoderma harzianum* strain B97	Fungicide	P
*Trichoderma harzianum* Rifai strains T‐22 and ITEM 908	Fungicide	A
*Trichoderma harzianum* strain T78	Fungicide	P
*Verticillium albo‐atrum* (formerly *Verticillium dahliae*) strain WCS850	Fungicide	A
Zucchini yellow mosaic virus ‐ weak strain	Fungicide	A

^a^ Information extracted from the current list of active substances available in the “EU Pesticide Substances Database” (https://ec.europa.eu/food/plant/pesticides/eu‐pesticides‐database/active‐substances/?event=search.as) in September 2022.

^b^ Active substances can only be approved as plant protection products if they fulfil the approval criteria in accordance with Regulation (EC) No 1107/200 of the European Parliament and of the Council concerning the placing of plant protectionproducts on the market.

^c^ Abbreviations used: A, approved; P, under evaluation and pending approval.

To address the legislative issues regarding the inability to use certain bacterial species as biocontrol agents, in a recent publication in *Microbial Biotechnology*, the possibility of heterologously expressing AGCs from *Burkholderia* species in *Paraburkolderia* strains was assessed (Petrova et al., 2022) – a recently re‐classified genus that mostly includes plant‐associated bacteria with biocontrol and bioremediation properties (Esmaeel et al., 2018). Although *Burkholderia* species are frequently isolated from plants and were shown to act as biocontrol agents (Mullins et al., 2019; Zhang et al., 2022), concerns about their potential pathogenic risks (French et al., 2020; Mannaa et al., 2018) have restricted their use as biopesticides. With the aim of engineering non‐pathogenic antibiotic producing strains with biocontrol abilities, Petrova and co‐workers constructed a novel vector to express AGC. This vector includes a yeast replication origin to take advantage of the in vivo recombinant capabilities of *Saccharomyces cerevisiae*. As a proof of concept, the AGC of the *Burkholderia* polyyne antibiotics cepacin A and caryophylli were cloned in this shuttle vector, which efficiently enabled the production of these bioactive metabolites in several *Paraburkolderia* strains. Given that the stability of these plasmids in *Paraburkolderia* strains required the presence of an antibiotic selective pressure (Petrova et al., 2022), further work will be focused on the development of novel synthetic biology techniques that allow the efficient integration of large biosynthetic gene clusters in the chromosome of heterologous hosts (Choi & Lee, 2020; Roux & Chooi, 2022). Taken together, these experimental approaches open exciting new opportunities for the development of biotechnological production platforms that allow the construction of *à la carte* biopesticide agents.

Current estimates indicate that agricultural production must increase by at least 60% by 2050 to sustain the growing food demand (Bhatta et al., 2021). Because of the urgent need to change current agricultural disease control strategies, current figures indicate an annual growth in global sales of biopesticides of more than 10% (Marrone, 2019). In addition, phage‐based biocontrol strategies are also being revisited (Holtappels et al., 2022). To broaden the spectrum of activity of microbial inoculants in agriculture and reduce the occurrence of resistance, current directions are focused on exploring consortia of biocontrol agents that are active against a broad range of plant pathogens and which exhibit different modes of action (Compant et al., 2019). A recent report in *Environmental Microbiology* (Yuan et al., 2022) proved the efficacy of consortia consisting of up to six bacterial strains for the biocontrol of agricultural diseases.

## AUTHOR CONTRIBUTIONS

**Amalia Roca:** Conceptualization (equal); project administration (equal); writing – original draft (equal); writing – review and editing (equal). **Miguel A. Matilla:** Conceptualization (equal); project administration (equal); writing – original draft (equal); writing – review and editing (equal).

## FUNDING INFORMATION

Spanish Ministry for Science and Innovation/Agencia Estatal de Investigación (PID2019‐103972GA‐I00, RYC2019‐026481‐I).

## CONFLICT OF INTEREST

The authors declare that there is no conflict of interest.
